# PDEPT: polymer-directed enzyme prodrug therapy

**DOI:** 10.1054/bjoc.2001.2026

**Published:** 2001-09

**Authors:** R Satchi, T A Connors, R Duncan

**Affiliations:** Centre for Polymer Therapeutics, The School of Pharmacy, University of London, 29–39 Brunswick Square, London, WCIN IAX, UK

**Keywords:** PDEPT, tumour targeting, polymer prodrugs, HPMA copolymer, polymer-enzyme conjugate

## Abstract

Polymer-directed enzyme prodrug therapy (PDEPT) is a novel two-step antitumour approach using a combination of a polymeric prodrug and polymer-enzyme conjugate to generate cytotoxic drug selectively at the tumour site. In this study the polymeric prodrug N-(2-hydroxypropyl) methacrylamide (HPMA) copolymer-Gly-Phe-Leu-Gly-doxorubicin conjugate PK1 (currently under Phase II clinical evaluation) was selected as the model prodrug, and HPMA copolymer-cathepsin B as a model for the activating enzyme conjugate. Following polymer conjugation (yield of 30–35%) HPMA copolymer-cathepsin B retained ~20–25% enzymatic activity in vitro. To investigate pharmacokinetics in vivo,^125^I-labelled HPMA copolymer-cathepsin B was administered intravenously (i.v.) to B16F10 tumour-bearing mice. HPMA copolymer-cathespin B exhibited a longer plasma half-life (free cathepsin B t_1/2α_= 2.8 h; bound cathepsin B t_1/2α_= 3.2 h) and a 4.2-fold increase in tumour accumulation compared to the free enzyme. When PK1 (10 mg kg^−1^dox-equiv.) was injected i.v. into C57 mice bearing subcutaneously (s.c.) palpable B16F10 tumours followed after 5 h by HPMA copolymer-cathepsin B there was a rapid increase in the rate of dox release within the tumour (3.6-fold increase in the AUC compared to that seen for PK1 alone). When PK1 and the PDEPT combination were used to treat established B16F10 melanoma tumour (single dose; 10 mg kg^−1^dox-equiv.), the antitumour activity (T/C%) seen for the combination PDEPT was 168% compared to 152% seen for PK1 alone, and 144% for free dox. Also, the PDEPT combination showed activity against a COR-L23 xenograft whereas PK1 did not. PDEPT has certain advantages compared to ADEPT and GDEPT. The relatively short plasma residence time of the polymeric prodrug allows subsequent administration of polymer-enzyme without fear of prodrug activation in the circulation and polymer-enzyme conjugates have reduced immunogenicity. This study proves the concept of PDEPT and further optimisation is warranted. © 2001 Cancer Research Campaign   http://www.bjcancer.com


British Journal of Cancer (2001) 85(7), 1070–1076
© 2001 Cancer Research Campaign
doi: 10.1054/ bjoc.2001.2026, available online at http://www.idealibrary.com on IDEAL

### PDEPT: polymer-directed enzyme prodrug therapy

### I. HPMA copolymer-cathepsin B and PK1 as a model combination

**R Satchi, TA Connors and R Duncan***

Centre for Polymer Therapeutics, The School of Pharmacy, University of London, 29–39 Brunswick Square, London WCIN IAX, UK

**Summary** Polymer-directed enzyme prodrug therapy (PDEPT) is a novel two-step antitumour approach using a combination of a polymeric prodrug and polymer-enzyme conjugate to generate cytotoxic drug selectively at the tumour site. In this study the polymeric prodrug N-(2-hydroxypropyl) methacrylamide (HPMA) copolymer-Gly-Phe-Leu-Gly-doxorubicin conjugate PK1 (currently under Phase II clinical evaluation) was selected as the model prodrug, and HPMA copolymer-cathepsin B as a model for the activating enzyme conjugate. Following polymer conjugation (yield of 30–35%) HPMA copolymer-cathepsin B retained ~20–25% enzymatic activity in vitro. To investigate pharmacokinetics in vivo, $^{125}$I-labelled HPMA copolymer-cathepsin B was administered intravenously (i.v.) to B16F10 tumour-bearing mice. HPMA copolymer-cathespin B exhibited a longer plasma half-life (free cathepsin B $t_{1/2\alpha}$ = 2.8 h; bound cathepsin B $t_{1/2\alpha}$ = 3.2 h) and a 4.2-fold increase in tumour accumulation compared to the free enzyme. When PK1 (10 mg kg$^{-1}$ dox-equiv.) was injected i.v. into C57 mice bearing subcutaneously (s.c.) palpable B16F10 tumours followed after 5 h by HPMA copolymer-cathepsin B there was a rapid increase in the rate of dox release within the tumour (3.6-fold increase in the AUC compared to that seen for PK1 alone). When PK1 and the PDEPT combination were used to treat established B16F10 melanoma tumour (single dose; 10 mg kg$^{-1}$ dox-equiv.), the antitumour activity (T/C%) seen for the combination PDEPT was 168% compared to 152% seen for PK1 alone, and 144% for free dox. Also, the PDEPT combination showed activity against a COR-L23 xenograft whereas PK1 did not. PDEPT has certain advantages compared to ADEPT and GDEPT. The relatively short plasma residence time of the polymeric prodrug allows subsequent administration of polymer-enzyme without fear of prodrug activation in the circulation and polymer-enzyme conjugates have reduced immunogenicity. This study proves the concept of PDEPT and further optimisation is warranted.
© 2001 Cancer Research Campaign http://www.bjcancer.com

**Keywords**: PDEPT; tumour targeting; polymer prodrugs; HPMA copolymer; polymer-enzyme conjugate

Cancer chemotherapy is often restricted by adverse systemic toxicity and the appearance of multidrug resistance. Improvements, particularly for the treatment of common solid tumours, have been difficult to realise (Connors, 1996) and as a result combination therapies designed to increase active drug concentration in the tumour have been developed; namely antibody-directed enzyme prodrug therapy (ADEPT) (Bagshawe, 1987; Melton et al, 1996) and viral/gene-directed enzyme prodrug therapy (V/GDEPT) (Ram et al, 1993). Here we describe polymer-directed enzyme prodrug therapy (PDEPT), a novel two-step antitumour approach combining a polymeric prodrug and polymer-enzyme to generate cytotoxic drug at the tumour site (Figure 1). Conceptually, PDEPT proposes initial administration of the polymeric prodrug to promote tumour targetting before administration of the activating polymer-enzyme conjugate.

Polymer-enzyme conjugates such as polyethylene glycol (PEG)-L-asparaginase (Oncaspar®) have already been developed to the market (Ho et al, 1986). PEGylation of proteins reduces their immunogenicity and prolongs circulation time (Delgado et al, 1992). Although a new departure in cancer chemotherapy, several polymer-drug conjugates are already in early clinical trial (reviewed in Duncan, 1992; Duncan et al, 1996; Brocchini and Duncan, 1999). These include the N-(2-hydroxypropyl)methacrylamide (HPMA) copolymer anticancer conjugates PK1 (FCE 28068) (Vasey et al, 1999), PK2 (FCE 28069) (Ferry et al, 1999) and PNU (166945) (Ten Bokkel Hunink et al, 1998) and a PEG-camptothecin conjugate (Pendri et al, 1998). Reduced toxicity and activity in chemotherapy refractory patients has been described. In phase I HPMA copolymer-doxorubicin (dox) (PK1) (Figure 2A) displayed a maximum tolerated dose of 320 mg/m$^2$ (Vasey et al, 1999) and also showed antitumour activity. Moreover the clinical pharmacokinetics (PK1 $t_{1/2\alpha}$ = 1.8 h with no dose dependency of clearance) were very similar to those reported in animals (Seymour et al, 1990). Combination of polymer-drug and polymer-enzyme conjugates can capitalise on the ability of both to target solid tumour tissue passively by a mechanism termed the enhanced permeability and retention (EPR) effect (Matsumura and Maeda, 1986). This occurs due to the poorly organised tumour vasculature (Dvorak et al, 1988; Skinner et al, 1990) resulting in 'enhanced permeability' towards circulating molecules. The lack of lymphatic drainage in tumour tissue leads to increased 'retention'. The aim of this study was to establish the feasibility of the

*Received 17 January 2001*
*Revised 22 June 2001*
*Accepted 25 June 2001*

*Correspondence to:* R Duncan

*Present address: Professor Ruth Duncan, Welsh School of Pharmacy, Cardiff University, Redwood Building, King Edward VII Avenue, Cardiff CF10 3XF, Wales.

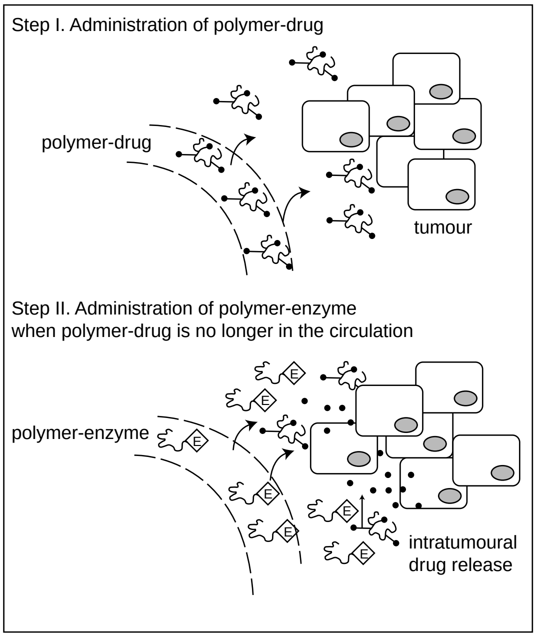

**Figure 1** Schematic representation of PDEPT

PDEPT concept using PK1 and HPMA copolymer-cathepsin B as a model combination. PK1 has proven ability to target solid tumours by the EPR effect (Seymour et al, 1994) with concomitant renal elimination resulting in low blood levels within 1–5 h in animals and man (Seymour et al, 1990; Vasey et al, 1999). HPMA copolymer-cathepsin B (Figure 2B) was selected for PK1 activation as the PK1 Gly-Phe-Leu-Gly polymer-dox linker was designed to permit intralysosomal dox liberation due to action of the lysosomal cysteine proteases (Duncan et al, 1984). First it was necessary to prepare an HPMA copolymer-cathepsin B conjugate that would retain sufficient enzyme activity after conjugation. Activity was monitored in vitro using a low molecular weight substrate N-Benzoyl-Phe-Val-Arg-p-nitroanilide hydrochloride (Bz-Phe-Val-Arg-NAp) and the polymeric substrate PK1. The biodistribution of $^{125}$I-labelled HPMA copolymer-cathepsin B and $^{125}$I-labelled cathepsin B was assessed in mice bearing subcutaneous (s.c.) B16F10 tumours and this model was also used to measure the kinetics of doxorubicin release after intravenous (i.v.) administration of PK1 alone (drug liberation by endogenous lysosomal enzymes) or PK1 followed, after 5 h, by HPMA copolymer-cathepsin B. Preliminary studies were conducted to establish the antitumour activity of PDEPT combination using the B16F10 model and a human non-small-cell lung carcinoma xenograft (COR-L23).

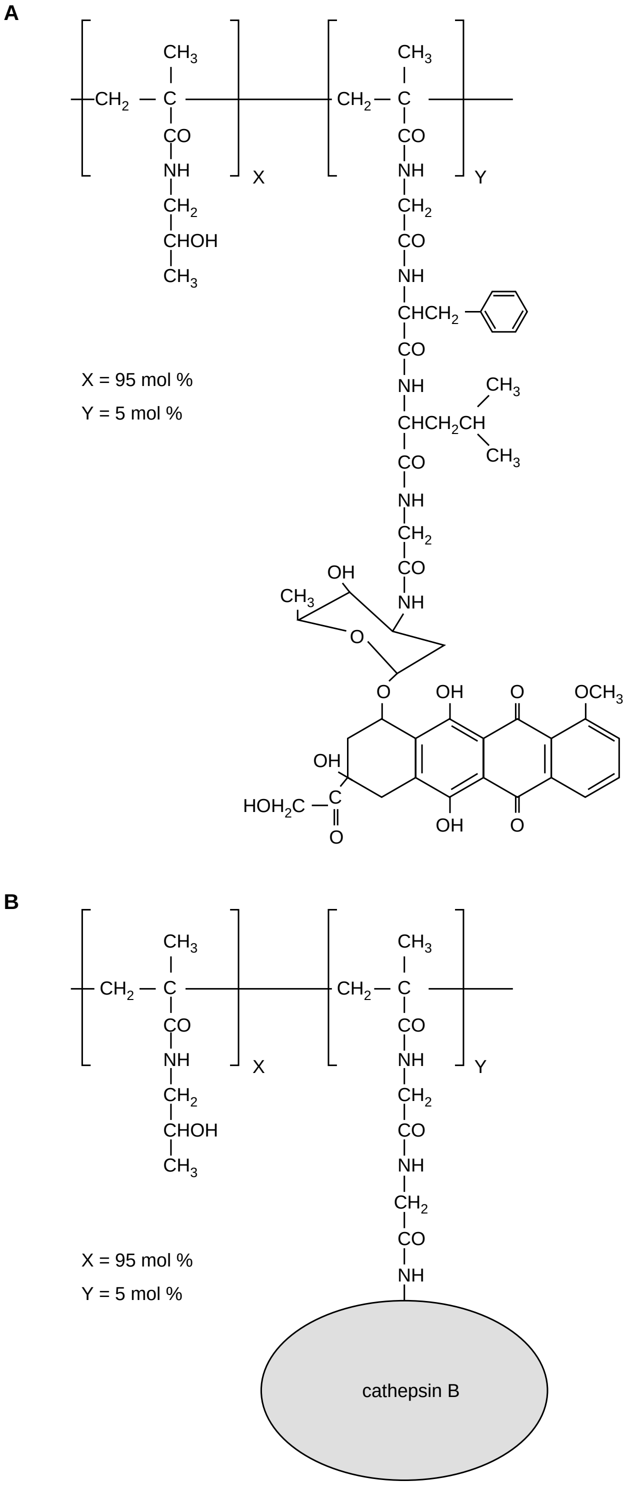

**Figure 2** Structures of (**A**) PK1 and (**B**) HPMA copolymer-Gly-Gly-cathepsin B

### MATERIALS AND METHODS

### Materials

Cathepsin B (EC 3.4.22.1) from bovine spleen, reduced glutathione, Bz-Phe-Val-Arg-NAp, EDTA, Cu(II) sulphate pentahydrate 4% w/v solution, bicinchoninic acid solution, barbitone buffer (B6632) were all purchased from Sigma (Dorset, UK). Doxorubicin hydrochloride (dox) was obtained from Aldrich Chem (UK). Daunomycin hydrochloride (dnm) was kindly donated by Rhone Poulence (France). Tris-HCl 0.5 M, pH 6.8, Tris-HCl 1.5 M, pH 8.8, sodium dodecyl sulphate (SDS) 10% (w/v), TEMED, 10% (w/v) ammonium persulfate, mercaptoethanol, bromophenol blue and 30% (w/v) 19:1 acrylamide/bis-acrylamide were purchased from Bio-Rad (UK). PK1 was synthesised as previously described (Rihova et al, 1989) and batches had the following characteristics: $M_w$ ~ 30,000 Da; $M_w/M_n$~1.3; 6–8% w/w of dox content. HPMA

© 2001 Cancer Research Campaign copolymer-Gly-Gly-p-nitrophenol (ONp) was obtained from Polymer Laboratories (Church Stretton, UK).

The cell line B16F10 murine melanoma was kindly donated by Prof Ian Hart (St Thomas' Hospital, London, UK). COR-L23 non-small-cell lung carcinoma human xenograft cell line was purchased from ECCAC (European Collection of Cell Cultures, Centre for Applied Biology, Microbiology and Research, Salisbury, Wiltshire, UK). Bantin and Kingman Ltd (Hull, UK) supplied the C57 black male mice, Balb/C male mice and nu/nu male mice.

### Synthesis and characterisation of HPMA copolymer-cathepsin B

Cathepsin B was bound to HPMA copolymer-Gly-Gly-ONp using a non-specific aminolysis reaction (Ulbrich et al, 1996). HPMA-Gly-Gly-ONp was dissolved in double distilled water (2 mg $ml^{-1}$) and the solution of cathepsin B (2 mg $ml^{-1}$) in 0.05 M phosphate buffer, pH 7.2, was added at 4˚C with stirring. The reaction mixture was stirred in the dark at pH 7.2 for 30 min. Then the pH was carefully raised during 4 h by adding saturated sodium tetraborate buffer up to pH 8.5 (to prevent enzyme denaturation). The mixture was stirred for another 4 h and the reaction was terminated by adding 1-amino-2-propanol (1/2 the equivalent related to the original ONp content). The final yellow solution was acidified by adding diluted HCl solution to pH 7.2. The conjugation reaction was followed by UV spectrophotometry to detect the release of ONp with time from the HPMA copolymer precursor (bound ONp $\lambda_{max}$ = 270 nm; free ONp $\lambda_{max}$ = 400 nm). To remove free polymer, free enzyme and other low molecular weight compounds, the conjugate was purified at first by centriprep-50 (cut-off 50 KDa) or dialysis membrane Spectra/POR CE (Cellulose Ester) sterile DispoDialyzer (Pierce & Warriner) MW cut-off 50 KDa. The crude reaction mixture and the HPMA copolymer-cathepsin B preparations were subject to SDS PAGE analysis to ensure purity and the yield of the reaction determined by BCA protein assay.

### Determination of cathepsin B activity

The in vitro activity of free and conjugated cathepsin B was first determined using the tripeptide substrate Bz-Phe-Val-Arg-NAp. Enzyme was incubated with substrate at 37˚C in citrate phosphate buffer (0.2 M, pH 5.5) containing EDTA (10 mM) and reduced glutathione (50 mM). NAp release was followed spectrophotometrically at 410 nm. Subsequently the ability of free and conjugated enzyme to cleave the high molecular weight substrate PK1 was assessed by HPLC analysis. PK1 was incubated with cathepsin B at 37˚C in a final volume of 1 ml: 400 $\mu$l of PK1 (1 mg $ml^{-1}$) in citrate buffer (pH 5.5, 0.2 M), 100 $\mu$l EDTA solution in buffer (10 mM), 100 $\mu$l reduced glutathione (GSH 50 mM) and 400 $\mu$l HPMA copolymer-cathepsin B or free cathepsin B in buffer (1 mg $ml^{-1}$). Samples (100 $\mu$l) were taken at various times, immediately frozen in liquid nitrogen and stored frozen in the dark until processed by HPLC (Wedge, 1990; Seymour et al, 1994) using daunomycin as internal standard (100 ng).

### Tumour models

All animal experiments were carried out according to the United Kingdom Co-ordinating Committee on Cancer Research (UKCCCR) guidelines for the welfare of animals in experimental neoplasia (UKCCCR guidelines, 1998).

### Body distribution of free and conjugated $^{125}$I-labelled-cathepsin B

Cathepsin B and HPMA-copolymer-Gly-Gly-cathepsin B were $^{125}$I-radiolabelled with Na[$^{125}$I] iodide using chloramine-T. The labelling efficiency and purity (<1% free [$^{125}$I]iodide) of the $^{125}$I-labelled products was then estimated using paper electrophoresis and the specific activity of each product was determined ($\mu$Ci $mg^{-1}$). Male C57BL/6J mice were inoculated s.c. with $10^5$ viable B16F10 cells. The tumour was allowed to establish until the area was approximately 50–70 $mm^2$ as measured by the product of 2 orthogonal diameters (*c*. 12 days). Free or conjugated cathepsin B (100 $\mu$l; $5 \times 10^5$ CPM) was injected into the tail vein of C57BL/6J mice ($n$ = 3 per time point). The mice were then placed in metabolic cages and killed at times up to 48 h. Blood samples were taken and organs dissected, weighed and homogenised in PBS. Triplicate samples (1 ml) were then assayed for radioactivity. The results were expressed as % dose injected. The blood volume of the mouse was calculated assuming 5.7 ml blood/100 g mouse (Dreyer and Ray, 1910).

### Evaluation of total and free doxorubicin after administration of PK1 and the PDEPT combination to mice bearing s.c. B16F10

Male C57BL/6J mice were inoculated s.c. with $10^5$ viable B16F10 cells. Animals bearing palpable B16F10 were injected i.v. with PK1 (10 mg $kg^{-1}$ dox-equiv.), or PK1 (10 mg $kg^{-1}$ dox-equiv.) followed after 5 h by free cathepsin B (3.63 mg $kg^{-1}$) or HPMA copolymer-Gly-Gly-cathepsin B (3.63 mg $kg^{-1}$ weight equiv. cathepsin B). They were killed at different time points up to 48 h post-PK1 injection. The following tissue samples were dissected: tumour, liver, kidneys, lung, heart, spleen, and urine and blood samples were collected. Samples were weighed, homogenised in PBS, mixed with 100 ng dnm. Total and free dox was analysed by HPLC (Wedge, 1990; Seymour et al, 1994).

### Antitumour activity in B16F10 and COR-L23 models

Mice (6–8 weeks) were inoculated s.c. with tumour cells ($10^5$ B16F10 cells into C57 mice or $10^6$ COR-L23 cells into nu/nu mice). When tumours became palpable (~25 $mm^2$) treatment was initiated. Groups ($n$ = 5) were injected i.v. with PK1 (10 mg $kg^{-1}$ dox-equiv.), or PK1 (10 mg $kg^{-1}$ dox-equiv.) followed after 5 h, by free cathepsin B (3.63 mg $kg^{-1}$) or HPMA copolymer-Gly-Gly-cathepsin B (3.63 mg $kg^{-1}$ weight equivalent of cathepsin B). Additional groups were treated with saline, free dox (10 mg $kg^{-1}$) or free cathepsin B (3.63 mg $kg^{-1}$). Animals were weighed and tumours measured daily. Mice were killed when the tumour reached or surpassed the size of 289 $mm^2$. The ratio (T/C) expressed as a percentage of the mean survival time for a treated group of animals (T) compared with the mean survival time of the control group (C) value was used to define antitumour activity. Statistical significance was assessed using the student's *t*-test for small samples and $P$ values < 0.05 were taken as statistically significant.

### RESULTS

HPMA copolymer-Gly-Gly-cathepsin B conjugate were prepared with a yield of 30–35% in respect of the bound protein. Whereas

 © 2001 Cancer Research Campaign

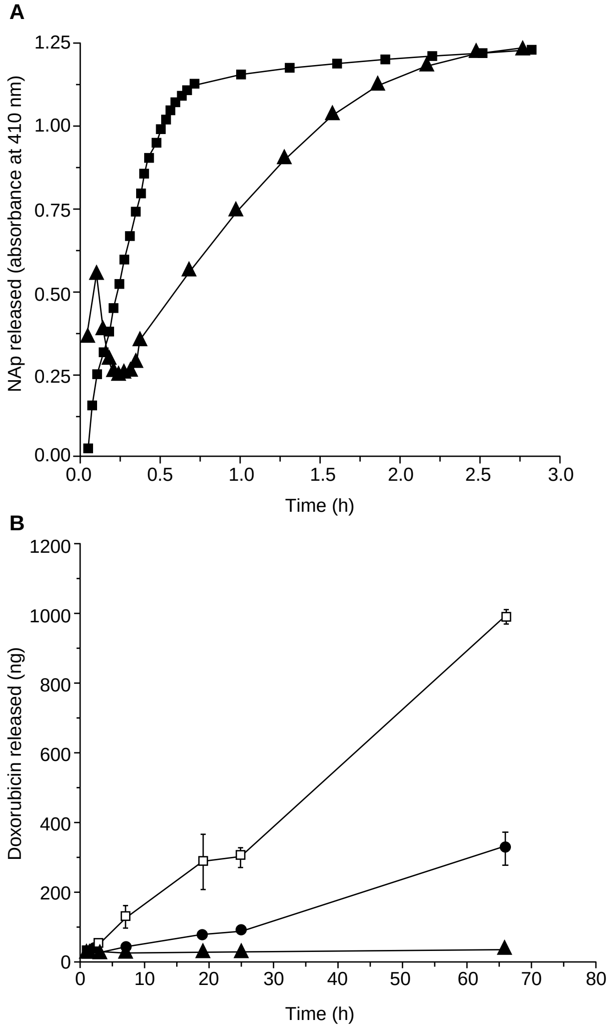

**Figure 3** Comparison of enzymatic activity of free cathepsin B and HPMA copolymer-cathepsin B in vitro. Panel (**A**) Activity against Bz-Phe-Val-Arg-NAp; cathepsin B (■) and HPMA copolymer-cathepsin B (▲) and panel (**B**) using PK1 as a substrate; cathepsin B (□); HPMA copolymer-cathepsin B (●). Release of dox from PK1 in the absence of enzyme is included as a control (▲). See the Methods section for experimental details free cathepsin B has a band at 30 kDa on SDS PAGE the HPMA copolymer-cathepsin B conjugates had a molecular weight in the range 60–97 kDa. After purification no free enzyme was detectable (results not shown). Against the Bz-Phe-Val-Arg-NAp substrate, HPMA copolymer-cathepsin B retained 24.4% the activity of free enzyme (Figure 3A) and with PK1 as substrate, the cathepsin B conjugate retained 19.5% enzyme activity (Figure 3B). The blood clearance of both the $^{125}$I-labelled enzyme and conjugate was biphasic. Clearance of $^{125}$I-labelled cathepsin B had a $t_{1/2\alpha}$ = 2.8 h and $t_{1/2\beta}$ = 8.9 h. The conjugate had a longer blood residence time ($t_{1/2\alpha}$ = 3.2 h and $t_{1/2\beta}$ = 9.3 h). The conjugate blood AUC was 1.3-fold greater than seen for free enzyme (Figure 4A) and the tumour AUC was 3.6-fold greater (Figure 4B).

After administration of PK1 to B16F10 melanoma-bearing mice the levels of free dox detected in tumour were constant with time at ~2500–5000 ng/tumour. When the HPMA copolymer-cathepsin B conjugate was administered 5 h after PK1 there was a marked increase in tumoural free dox levels (Figure 5). Over the 5–48 h period this caused a 3.6-fold increase in the free dox AUC

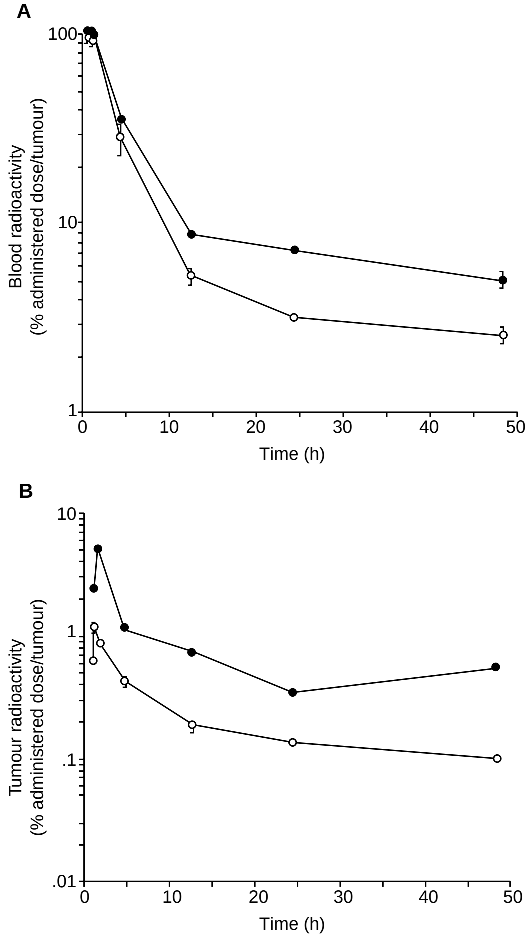

**Figure 4** Body distribution of $^{125}$I-labelled cathepsin B (○) and $^{125}$I-labelled HPMA copolymer cathepsin B (●) in mice bearing s.c. B16F10 tumours. Panel (**A**) blood clearance of radioactivity and panel (**B**) tumour levels of radioactivity. Values represent the mean ± SE (*n* = 3)

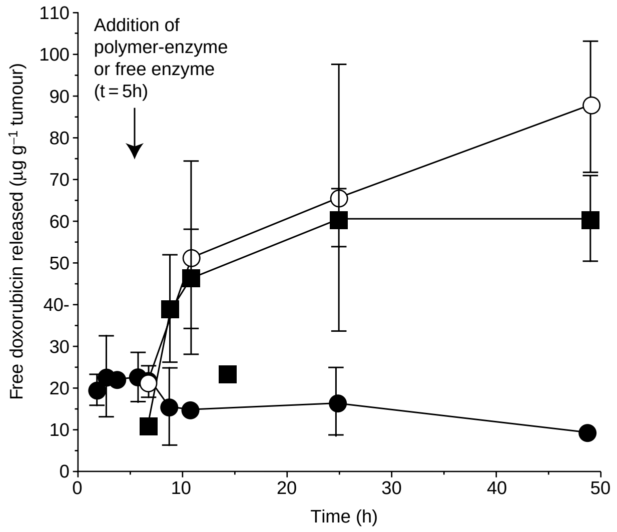

**Figure 5** Levels of dox in B16F10 tumours after treatment with PK1 or the PDEPT combination. The data shown represent free doxorubicin levels after administration at time zero of PK1 (●), PK1 followed by HPMA copolymer-cathepsin B at 5 h (■) and PK1 followed by cathepsin B at 5 h (○). Whereas HPMA copolymer-cathepsin and free enzymes were given at the same enzyme dose (3.63 mg kg$^{-1}$) the free enzyme is ~ 5 × more active. Values represent the mean ± SE (*n* = 3)

**Table 1** Antitumour activity of PK1 and the PDEPT model administered i.v. to C57 mice bearing s.c. B16F10 murine melanoma

| Treatment | Dose (mg kg$^{-1}$) | Time to progression (Days ± SE) | T/C (%) | No. of toxic deaths |
|---|---|---|---|---|
| Saline | – | 6.8 ± 0.5 | 100 | 0/5 |
| Doxorubicin | 10 | 9.8 ± 0.9 | 144* | 0/5 |
| Cathepsin B | 3.63 | 8.4 ± 1.4 | 123$^{NS}$ | 0/5 |
| PK1 + cathepsin B | 10 | 9.2 ± 0.8 | 135* | 0/5 |
| PK1 | 10 | 10.33 ± 0.3 | 152** | 0/5 |
| PK1 + HPMA-cathepsin B | 10 | 11.4 ± 1.2 | 168** | 0/5 |

Levels of significance * $P \leq 0.05$, ** $P \leq 0.01$, NS = no significant difference.

**Table 2** Antitumour activity of PK1 and the PDEPT model administered i.v. to nu/nu mice bearing s.c. COR-L23 non-small cell lung carcinoma

| Treatment | Dose (mg kg$^{-1}$) | Time to progression (Days ± SE) | T/C (%) | No. of toxic deaths |
|---|---|---|---|---|
| Saline | | 19.6 ± 1.3 | 100 | 0/5 |
| Doxorubicin | 10 | 25.4 ± 1.6 | 129* | 0/5 |
| PK1 | 10 | 22.6 ± 1.4 | 115$^{NS}$ | 0/5 |
| PK1 + HPMA-cathepsin B | 10 | 24.8 ± 1.1 | 127* | 0/5 |

Levels of significance * $P \leq 0.03$, NS = no significant difference.

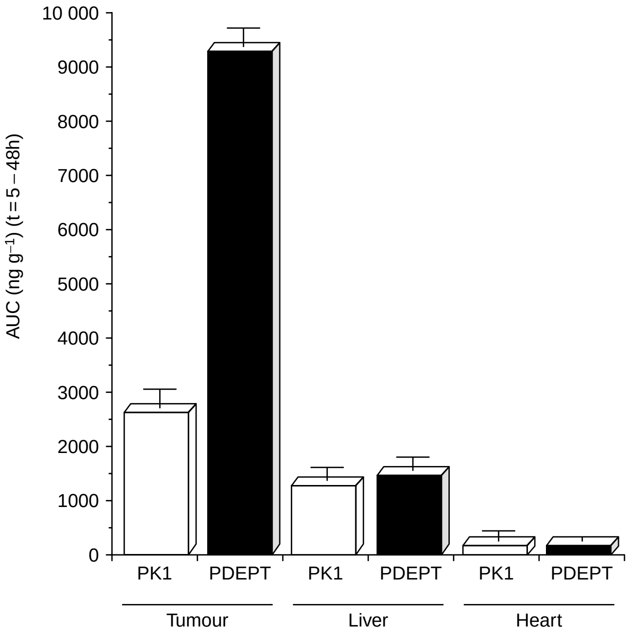

**Figure 6** Free dox levels (AUC) measured in tumour and normal tissues after treatment of C57 mice bearing B16F10 s.c. tumours with either PK1 or the PDEPT combination compared to that seen after administration of PK1 alone (Figure 5). Administration of free cathepsin B at the same protein dose (3.63 protein mg kg$^{-1}$; ~5 times the enzyme activity) resulted in an equivalent enhancement of dox liberation (results not shown). Little free dox (< less than 10 ng) was detectable in the blood at any time point (Figure 5) confirming that at 5 h after PK1 administration, no PK1 was available in the circulation to act as a substrate for the administered enzymes. Whereas, administration of HPMA copolymer-cathepsin B led to increased levels of free dox in tumour tissue there was no significant increase in dox levels in normal tissues (Figure 6).

A dox-equiv. dose of 10 mg kg$^{-1}$ was chosen for all the treatments used in preliminary experiments investigating the antitumour activity of the PDEPT combination. Mice bearing s.c. B16F10 showed increased survival when treated with free dox, PK1 and the PDEPT combination (Table 1). Cathepsin B alone was not active. During this study there were neither toxic deaths nor animal weight loss (results not shown). A significant decrease (compared with control mice group) was observed in tumour growth rate in those animals treated with dox, PK1, and the PDEPT combination (Figure 7). Nude mice bearing COR-L23 showed increased survival when treated with dox alone and the PDEPT combination, although PK1 was not active in this model at the dose used (Table 2). A significant decrease in the tumour growth rate was observed after treatment of COR-L23 with either dox or the PDEPT combination (Figure 8).

### DISCUSSION

Although ADEPT and V/GDEPT have demonstrated improved therapeutic activity in animal models and pilot human studies (Bagshawe et al, 1995; Springer and Niculesco-Duvaz, 1996), a number of inherent limitations have emerged. Antibody-enzyme conjugates are often highly immunogenic (Sharma et al, 1992) and individualised constructs are needed for tumours expressing different antigens. The long plasma half-life of antibody-enzyme leads to difficulties in optimisation of the dosing schedule for subsequently administered prodrug. In some cases a second clearing antibody has been required to prevent non-specific prodrug activation in the circulation. V/GDEPT allows combination of oncogene targeting with chemotherapy, but it has inherent problems associated with viral vectors, and the low and heterogeneous enzyme expression in vivo (Connors, 1995; McNeish et al, 1997). As the duration and reproducibility of enzyme expression is difficult to evaluate on an individual patient basis, optimisation of the schedule for prodrug administration again may be difficult. PDEPT offers a number of advantages in comparison with ADEPT and V/GDEPT HPMA copolymer-protein conjugates

 © 2001 Cancer Research Campaign

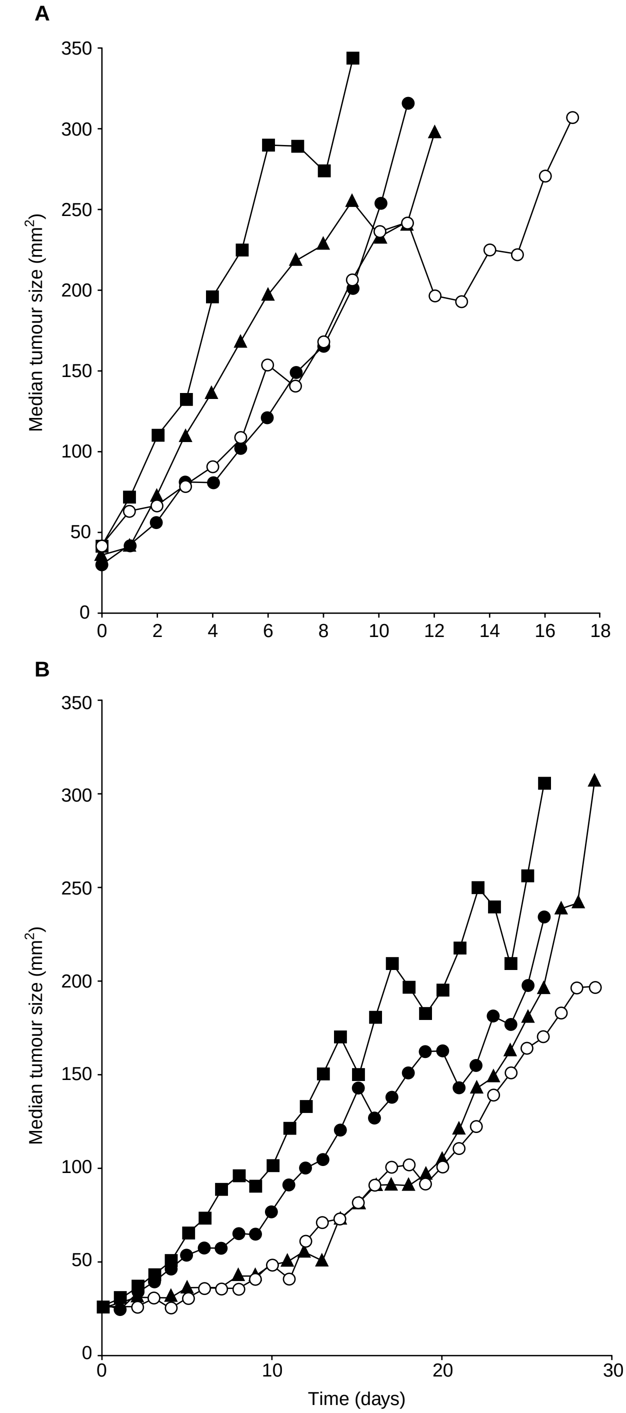

**Figure 7** Tumour size after treatment with dox, PK1, cathepsin B or the PDEPT combination. Panel (**A**) shows B16F10 tumours and Panel (**B**) COR-L23 tumours. For both the key is saline (■), dox (10 mg kg$^{-1}$) (▲), PK1 (10 mg kg$^{-1}$ dox-equiv.) (●) and PK1 (10 mg kg$^{-1}$ dox-equiv.) + HPMA copolymer-cathepsin B (3.63 mg kg$^{-1}$ protein-equiv) (○). Values represent the median tumour size

(like PEGylated proteins) display little or no immunogenicity (Flanagan et al, 1990). Additionally, the opportunity to administer a polymeric prodrug first (clinical pharmacokinetics has confirmed rapid plasma elimination whilst retaining the possibility of tumour targeting) can circumvent the problems associated with premature prodrug activation in the circulation.

Using the model combination of PK1 and HPMA copolymer-cathepsin B, this study has confirmed the feasibility of the 3 key aspects of the PDEPT concept. (1) Synthesis of a polymer-enzyme conjugate that retains activity against a polymeric prodrug, (2) tumour targeting of both components by the EPR effect and (3) spatial accessibility of polymer-enzyme to the polymeric prodrug in the tumour interstitium. Synthesis of antibody-and polymer-enzyme conjugates is often problematic. Conjugation typically has low yield (10–15%) (Svensson et al, 1994; Melton et al, 1996) and results in reduced enzyme activity. Ashihara et al (1978) showed that the activity of PEG-L-asparaginase conjugates decreased dramatically with increasing PEG molecular weight and in proportion to the degree of PEG substitution. The semi-random aminolysis method used to bind HPMA copolymer precursor to cathepsin B resulted in conjugates with a high yield (30–35% protein conjugation) and also conjugates with retained enzyme activity in vitro against both the low molecular weight and macromolecular substrates (20–25%) (Figure 3).

$^{125}$I-Labelled cathepsin B had a longer half-life ($t_{1/2\alpha}$ = 2.8 h) anticipated from the values reported for other (mainly bacterial) enzymes. This may be explained by the homology of the bovine and the mouse cathepsin B. None the less, the $^{125}$I-labelled HPMA copolymer-cathepsin B conjugate did show a longer blood circulation (Figure 4A) consistent with the reduced cellular clearance and/or increased resistance to proteolysis (Francis et al, 1992). Increased circulation time led to improved conjugate tumour probably attributable to passive targeting by the EPR effect.

Using the B16F10 model, Seymour et al (1994) showed that i.v. administration of PK1 produced a total doxorubicin AUC that was ~17-fold higher than seen for free dox at equi-dose (5 mg kg$^{-1}$). It was also noted that dox liberation had a lag phase of 30–60 min and thereafter the free doxorubicin levels plateaued. The same basic pattern of PK1 doxorubicin release was observed here (Figure 5). However, subsequent administration of HPMA copolymer-cathepsin B (Figure 5) clearly led to a marked increase in PK1 doxorubicin liberation. This observation confirms both extracellular accessibility of the polymer enzyme to the polymer prodrug and verifies retention of the conjugate enzyme activity in vivo. Administration of free cathepsin B (the same protein dose but ~5 times the enzyme activity) also led to intratumoural release of doxorubicin from PK1 (Figure 5). The apparently greater potency of the polymer-enzyme conjugate in this experiment was simply consistent with the improved EPR-mediated targeting of the conjugate compared with to free enzyme.

It was considered important to ensure that the PDEPT combination would produce antitumour activity. Although the preliminary studies reported here are limited – using a single treatment and the same doxorubicin dose as used for the pharmacokinetic studies (10 mg kg) – they verify activity of the PDEPT combination in both the B16F10 and COR-L23 tumour models. Previous studies using the ADEPT system in a CC3 human choriocarcinoma xenograft (Springer et al, 1991) clearly showed that antitumour activity improved dramatically following optimisation of both schedule and dose of the antibody-enzyme conjugate and the prodrug. Future experiments with the HPMA copolymer-cathepsin B and PK1 model combination will optimise both HPMA copolymer-enzyme and PK1 doses and also the length of the repeated cycle of administration. As we understand more of the clinical profile of PK1 in the ongoing phase II studies it may be clinically interesting to consider an activating enzymes as an adjunct to this therapy. However, other PDEPT combinations involving non-mammalian

*© 2001 Cancer Research Campaign* 

enzymes are currently considered priority for development and these include an HPMA copolymer-β-lactamase and a related polymeric prodrug (Satchi et al, 1999).

### ACKNOWLEDGEMENTS

We would like to thank The British Council, The Overseas Research Students Awards Scheme, The Laura de Saliceto University Postgraduate Trust Studentship and The Wingate Fellowship for financial support.

### REFERENCES

Ashihara Y, Kono T, Yamazaki S and Inada Y (1978) Modification of *E. coli* L-asparaginase with polyethylene glycol: disappearance of binding ability to anti-asparaginase serum. *Biochem Biophys Res Commun* **83**: 385–391

Bagshawe KD (1987) Antibody directed enzymes revive anti-cancer prodrugs concept. *Br J Cancer* **56**: 531–532

Bagshawe KD, Sharma SK, Springer CJ and Antoniw P (1995) Antibody directed enzyme prodrug therapy (ADEPT): a pilot scale clinical trial. *Tumour Target* **1**: 17–29

Baillie-Johnson H, Twentyman PR, Fox NE, Walls GA, Workman P, Watson JV, Johnson N, Reeve JG and Bleehen NM (1985) Establishment and characterisation of cell lines from patients with lung cancer (predominantly small cell carcinoma). *Br J Cancer* **52**: 495–504

Brocchini S and Duncan R (1999) Polymer drug conjugates: drug release from pendent linkers. In: *Encyclopaedia of Controlled Release* (Ed. E Mathiovitz), 786–816 Wiley: New York

Connors TA (1995) The choice of prodrugs for gene directed enzyme prodrug therapy of cancer. *Gene Therapy* **2**: 702–709

Connors TA (1996) Is there a future for cancer chemotherapy? *Ann Oncol* **7**: 445–452

Delgado C, Francis GE and Fisher D (1992) The uses and properties of PEG-linked proteins. *Crit Rev Therap Drug Carrier Sys* **9**: 249–304

Dreyer G and Ray W (1910) III The blood volume of mammals as determined by experiments upon rabbits, guinea-pigs and mice and its relationship to body weight and surface area expressed in a formula. Philosophical Transactions of The Royal Society for Science London, Section B **201**: 133–137

Duncan R (1992) Drug-polymer conjugates: potential for improved chemotherapy. *Anticancer drugs* **3**: 175–210

Duncan R (1999) Polymer conjugates for tumour targeting and intracytoplasmic delivery. The EPR effect as a common gateway? *Pharm Sci and Technol Today* **2**: 441–449

Duncan R, Cable HC, Lloyd JB, Rejmanova P and Kopecek J (1984) Polymers containing enzymatically degradable bonds, 7. Design of oligopeptide side chain in poly N-(2-hydroxypropyl)methacrylamide copolymers to promote efficient degradation by lysosomal enzymes. *Makromol Chem* **184**: 1997–2008

Duncan R, Dimitrijevic S and Evagorou EG (1996) The role of polymer conjugates in the treatment and diagnosis of cancer. *S.T.P. Pharma Sci* **6**(4): 237–263

Dvorak HF, Nagy JA, Dvorak JT and Dvorak AM (1988) Identification and characterisation of the blood vessels of solid tumours that are leaky to circulating macromolecules. *Am J Pathol* **133**: 95–109

Flanagan PA, Rihova B, Subr V, Kopecek J and Duncan R (1990) Immunogenicity of protein-N-(2-hydroxypropyl)methacrylamide copolymer conjugates measured in A/J and B10 mice. *J Bioact Compat Polymers* **5**: 151–166

Ferry DR, Seymour LW, Anderson D, Hesselwood S, Julyan P, Poyner R, Guest P, Doran J and Kerr DJ (1999) Phase I trial of liver targeted HPMA copolymer doxorubicin PK2, pharmacokinetics, spect imaging of $^{123}$I-PK2 and activity in hepatoma. Proceedings of the American Society for Clinical Oncology **21**: 4–13

Francis GE, Delgado C and Fisher D (1992) PEG-modified proteins, In: Stability of proteins pharmaceuticals (Part B): *In vivo* pathways of degradation and strategies for protein stabilisation, TJ Ahem and MC Manning (Eds.), New York, Plenum Press, 235–263

Ho DH, Brown NS, Yen A, Holmes R, Keating M, Abuchowski A, Newman RA and Krakoff IH (1986) Clinical pharmacology of polyethylene glycol-L-asparaginase. *Drug Metabol Dispos* **14**: 349–352

Matsumura Y and Maeda H (1986) A new concept for macromolecular therapeutics in cancer chemotherapy: mechanism of tumour tropic accumulation of proteins and antitumour agent SMANCS. *Cancer Res* **46**: 6387–6392

McNeish IA, Searle PF, Young LS and Kerr DJ (1997) Gene directed enzyme prodrug therapy for cancer. *Adv Drug Del Rev* **26**: 173–184

Melton RG, Knox RJ and Connors TA (1996) Antibody-Directed Enzyme Prodrug Therapy (ADEPT). *Drugs of The Future* **21**(2): 167–181

Muggia FM (1999) Doxorubicin-polymer conjugates: further demonstration of the concept of enhanced permeability and retention. *Clin Cancer Res* **5**: 7–8

Pendri A, Conover CD and Greenvald RB (1998) Antitumour activity of paclitaxel-2-glycinate conjugated to poly(ethylene glycol): a water soluble prodrug. *Anti-Cancer Drug Design* **13**: 387–395

Ram Z, Culver KW, Walbridge S, Blaese RM and Oldfield EH (1993) In situ retroviral-mediated gene transfer for the treatment of brain tumours in rats. *Cancer Res* **53**: 83–88

Rihova B, Bilej M, Vetvicka V, Ulbrich K, Strohalm J, Kopecek J and Duncan R (1989) Biocompatibility of N-(2-hydroxypropyl)methacrylamide copolymers containing adriamycin. Immunogenicity, effect of haematopoietic stem cells in bone marrow in vivo and effect on mouse splenocytes and human peripheral blood lymphocytes in vitro. *Biomaterials* **10**: 335–342

Satchi R and Duncan R (1998) PDEPT: Polymer Directed Enzyme Prodrug Therapy-*in vitro* and *in vivo* characterisation. Proceedings of the NCI-EORTC Symposium on New Drugs in Cancer Therapy **10**: 83

Seymour LW, Ulbrich K, Strohalm J, Kopecek J and Duncan R (1990) The pharmacokinetics of polymer-bound adriamycin. *Biochem Pharmacol* **39**(6): 1125–1131

Seymour LW, Ulbrich K, Styger PS, Brereton M, Subr V, Strohalm J and Duncan R (1994) Tumour tropism and anti-cancer efficacy of polymer-based doxorubicin prodrugs in the treatment of subcutaneous murine B16F10 melanoma. *Br J Cancer* **70**: 636–641

Sharma SK, Bagshawe KD, Melton RG and Sherwood RF (1992) Human immune response to monoclonal antibody-enzyme conjugates in ADEPT pilot clinical trial. *Cell Biophys* **21**: 109–120

Skinner A, Tutton PJM and O'Brien PE (1990) Microvascular architecture of experimental colon tumours in the rat. *Cancer Res* **50**: 2411–2417

Springer CJ and Niculesco-Duvaz I (1996) Gene-directed enzyme prodrug therapy (GDEPT): choice of prodrugs. *Adv Drug Del Rev* **22**: 351–364

Springer CJ, Bagshawe KD, Sharma SK, Searle F, Boden JA, Antoniw P, Burke PJ, Rogers GT, Sherwood RF and Melton RG (1991) Ablation of human choriocarcinoma xenografts in nude mice by antibody-directed enzyme prodrug therapy (ADEPT) with three novel compounds. *Eur J Cancer* **27** (11): 1361–1366

Svensson PH, Wallace MP and Senter P (1994) Synthesis and characterisation of monoclonal antibody-β-lactamase conjugates. *Bioconjugate Chem* **5**: 262–267

Ten Bokkel Hunink WW, Terwogt JM, Dubbelman R, Valkenet L, Zurlo MG, Schellens JHM and Beijnen JH (1998) Phase I and pharmacokinetics study of PNU 166945, a polymer formulated paclitaxel. *3rd Int Symp Polymer Therap* 12

Ulbrich K, Strohalm J, Subr V, Plocova D, Duncan R and Rihova B (1996) Polymeric conjugates of drugs and antibodies for site-specific drug delivery. *Macromol Symp* **103**: 177–192

United Kingdom Co-ordinating Committee on Cancer Research (UKCCR) guidelines for the welfare of animals in experimental neoplasia (Second Edition) (1998) *Br J Cancer* **77**(1): 1–10

Vasey PA, Kaye SB, Morrison R, Twelves C, Wilson P, Duncan R, Thomson AH, Murray LS, Hilditch TE, Murray T, Burtles S, Fraier D, Frigerio E and Cassidy J (1999) Phase I clinical and pharmacokinetic study of PK1 [N-(2-hydroxypropyl) methacrylamide copolymer doxorubicin]: first member of a new class of chemotherapeutic agents-drug-polymer conjugates. *Clin Cancer Res* **5**: 83–94

Wedge SR (1990) Mechanism of action of action of polymer anthracyclines: potential to overcome multidrug resistance. Ph.D. Thesis, Keele University 49–64

 *© 2001 Cancer Research Campaign*

## References

[PDF_00672] Ashihara Y., Kono T., Yamazaki S., Inada Y. (1978). Modification of E. coli L-asparaginase with polyethylene glycol: disappearance of binding ability to anti-asparaginase serum.. Biochem Biophys Res Commun.

[PDF_00675] Bagshawe K. D. (1987). Antibody directed enzymes revive anti-cancer prodrugs concept.. Br J Cancer.

[PDF_00680] Baillie-Johnson H., Twentyman P. R., Fox N. E., Walls G. A., Workman P., Watson J. V., Johnson N., Reeve J. G., Bleehen N. M. (1985). Establishment and characterisation of cell lines from patients with lung cancer (predominantly small cell carcinoma).. Br J Cancer.

[PDF_00689] Connors T. A. (1996). Is there a future for cancer chemotherapy? The Michel Clavel Lecture.. Ann Oncol.

[PDF_00687] Connors T. A. (1995). The choice of prodrugs for gene directed enzyme prodrug therapy of cancer.. Gene Ther.

[PDF_00691] Delgado C., Francis G. E., Fisher D. (1992). The uses and properties of PEG-linked proteins.. Crit Rev Ther Drug Carrier Syst.

[PDF_00697] Duncan R. (1992). Drug-polymer conjugates: potential for improved chemotherapy.. Anticancer Drugs.

[PDF_00699] Duncan R (1999). Polymer conjugates for tumour targeting and intracytoplasmic delivery. The EPR effect as a common gateway?. Pharm Sci Technolo Today.

[PDF_00709] Dvorak H. F., Nagy J. A., Dvorak J. T., Dvorak A. M. (1988). Identification and characterization of the blood vessels of solid tumors that are leaky to circulating macromolecules.. Am J Pathol.

[PDF_00725] Ho D. H., Brown N. S., Yen A., Holmes R., Keating M., Abuchowski A., Newman R. A., Krakoff I. H. (1986). Clinical pharmacology of polyethylene glycol-L-asparaginase.. Drug Metab Dispos.

[PDF_00731] Kerr DJ, Young LS, Searle PF, McNeish IA (1997). Gene directed enzyme prodrug therapy for cancer.. Adv Drug Deliv Rev.

[PDF_00728] Matsumura Y., Maeda H. (1986). A new concept for macromolecular therapeutics in cancer chemotherapy: mechanism of tumoritropic accumulation of proteins and the antitumor agent smancs.. Cancer Res.

[PDF_00735] Muggia F. M. (1999). Doxorubicin-polymer conjugates: further demonstration of the concept of enhanced permeability and retention.. Clin Cancer Res.

[PDF_00737] Pendri A., Conover C. D., Greenwald R. B. (1998). Antitumor activity of paclitaxel-2'-glycinate conjugated to poly(ethylene glycol): a water-soluble prodrug.. Anticancer Drug Des.

[PDF_00740] Ram Z., Culver K. W., Walbridge S., Blaese R. M., Oldfield E. H. (1993). In situ retroviral-mediated gene transfer for the treatment of brain tumors in rats.. Cancer Res.

[PDF_00743] Rihova B., Bilej M., Vetvicka V., Ulbrich K., Strohalm J., Kopecek J., Duncan R. (1989). Biocompatibility of N-(2-hydroxypropyl) methacrylamide copolymers containing adriamycin. Immunogenicity, and effect on haematopoietic stem cells in bone marrow in vivo and mouse splenocytes and human peripheral blood lymphocytes in vitro.. Biomaterials.

[PDF_00754] Seymour L. W., Ulbrich K., Steyger P. S., Brereton M., Subr V., Strohalm J., Duncan R. (1994). Tumour tropism and anti-cancer efficacy of polymer-based doxorubicin prodrugs in the treatment of subcutaneous murine B16F10 melanoma.. Br J Cancer.

[PDF_00751] Seymour L. W., Ulbrich K., Strohalm J., Kopecek J., Duncan R. (1990). The pharmacokinetics of polymer-bound adriamycin.. Biochem Pharmacol.

[PDF_00758] Sharma S. K., Bagshawe K. D., Melton R. G., Sherwood R. F. (1992). Human immune response to monoclonal antibody-enzyme conjugates in ADEPT pilot clinical trial.. Cell Biophys.

[PDF_00761] Skinner S. A., Tutton P. J., O'Brien P. E. (1990). Microvascular architecture of experimental colon tumors in the rat.. Cancer Res.

[PDF_00765] Springer C. J., Bagshawe K. D., Sharma S. K., Searle F., Boden J. A., Antoniw P., Burke P. J., Rogers G. T., Sherwood R. F., Melton R. G. (1991). Ablation of human choriocarcinoma xenografts in nude mice by antibody-directed enzyme prodrug therapy (ADEPT) with three novel compounds.. Eur J Cancer.

[PDF_00771] Svensson H. P., Wallace P. M., Senter P. D. (1994). Synthesis and characterization of monoclonal antibody-beta-lactamase conjugates.. Bioconjug Chem.

[PDF_00783] Vasey P. A., Kaye S. B., Morrison R., Twelves C., Wilson P., Duncan R., Thomson A. H., Murray L. S., Hilditch T. E., Murray T. (1999). Phase I clinical and pharmacokinetic study of PK1 [N-(2-hydroxypropyl)methacrylamide copolymer doxorubicin]: first member of a new class of chemotherapeutic agents-drug-polymer conjugates. Cancer Research Campaign Phase I/II Committee.. Clin Cancer Res.

